# *Antrodia camphorata*-Derived Antrodin C Inhibits Liver Fibrosis by Blocking TGF-Beta and PDGF Signaling Pathways

**DOI:** 10.3389/fmolb.2022.835508

**Published:** 2022-02-15

**Authors:** Xin-Yi Xu, Yan Geng, Hao-Xiang Xu, Yilin Ren, Deng-Yang Liu, Yong Mao

**Affiliations:** ^1^ Institute of Cancer, Affiliated Hospital of Jiangnan University, Wuxi, China; ^2^ School of Life Science and Health Engineering, Jiangnan University, Wuxi, China; ^3^ Department of Urology, Affiliated Wuxi No. 2 Hospital of Nanjing Medical University, Wuxi, China; ^4^ Department of Gastroenterology, Affiliated Hospital of Jiangnan University, Wuxi, China; ^5^ Department of Oncology, Affiliated Hospital of Jiangnan University, Wuxi, China

**Keywords:** liver disease, cell migration, hepatic stellate cells, extracellular matrix, cell signaling

## Abstract

Hepatic stellate cells (HSCs) play an essential role in the development of liver fibrosis. *Antrodia camphorata* (*A. camphorata*) is a medicinal fungus with hepatoprotective effect. This study investigated whether Antrodin C, an *A. camphorata*-fermented metabolite, could exert a protective role on liver fibrosis both *in vitro* and *in vivo*. The anti-fibrotic effect of Antrodin C was investigated in CFSC-8B cell (hepatic stellate cell) stimulated by transforming growth factor-β1 (TGF-β1) or platelet-derived growth factor-BB (PDGF-BB) *in vitro* and in CCl_4_ induced liver fibrosis in mice. Antrodin C (50 μM) inhibited TGF-β1 or PDGF-BB stimulated CFSC-8B cell activation, migration and extracellular matrix (ECM) accumulation (all *p* < 0.05). Antrodin C (3, 6 mg/kg/d) oral administration reduced the degree of liver fibrosis induced by CCl_4_ in mice. Antrodin C down-regulated the expression of α-smooth muscle actin (α-SMA) and collagen I in fibrotic livers. Furthermore, Antrodin C ameliorated alanine aminotransferase (ALT) and aspartate aminotransferase (AST) elevation in serum (all *p* < 0.05). Mechanistically, Antrodin C executes its anti-fibrotic activity through negatively modulate TGF-β1 downstream SMAD Family Member 2 (Smad2), AKT Serine/Threonine Kinase 1 (AKT), extracellular signal-regulated kinase (ERK), and P38 MAP Kinase (P38), as well as PDGF-BB downstream AKT and ERK signaling pathways. Antrodin C ameliorates the activation, migration, ECM production in HSCs and CCl_4_-induced liver fibrosis in mice, suggesting that Antrodin C could serve as a protective molecule against liver fibrosis.

**GRAPHICAL ABSTRACT:**
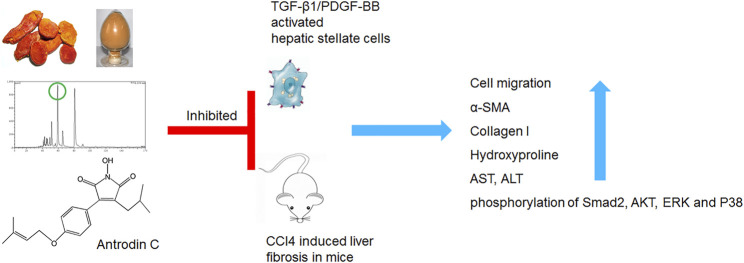


## Introduction

Organ fibrosis refers to the excessive deposition of extracellular matrix (ECM) in response to chronic tissue injury (Caligiuri et al., 2021). Hepatic fibrosis is caused by various factors, such as genomic mutations, toxic chemicals, hepatitis B/C, excessive alcohol consumption, and nonalcoholic steatohepatitis (Guerra et al., 2016; George et al., 2019). Liver fibrosis are wound-healing responses generated against an insult to the liver that cause liver injury (Aydin and Akcali, 2018). Liver fibrosis has the potential to progress to cirrhosis, liver cancer, and liver failure and its complications represent a massive health care burden worldwide (Deng et al., 2020). Aging has been considered as a risk factor for progression of fibrosis in hepatitis C and for poor outcome in alcoholic hepatitis (Goldstein et al., 2005; David and George, 2008). Therefore, it has been suggested that aging increases the susceptibility of liver fibrosis. Recent studies suggest that hepatic fibrosis could be reversible, however, its underlying mechanism remains uncertain and efficient anti-fibrotic drugs are urgently needed (Tan et al., 2021).

Hepatic stellate cells (HSCs) constitute the major mesenchymal cell type of the liver and play pivotal roles in a hepatic injury response (Dhar et al., 2020). Upon chronic liver injury, the quiescent HSCs (qHSCs) receive secreted signals and become activated HSCs (aHSCs) which express α-smooth muscle actin (α-SMA) and produce excessive extracellular matrix (ECM), including collagens and fibronectin (Jin et al., 2020; Yoon et al., 2020; Trivedi et al., 2021). Among many aberrant signaling molecules, transforming growth factor β1 (TGF-β1) and platelet-derived growth factor (PDGF) mediated signaling plays prominent roles in the transition of qHSCs into aHSCs (Kang et al., 2013).

*Antrodia camphorata* (also known as *Taiwanofungus camphoratus*, *Antrodia cinnamomea*) has been used as a traditional medicine or functional food for a long history in China for treating diarrhea, viral infection, diabetes mellitus liver cirrhosis, hepatoma and more (Zhenwei et al., 2021). Fermented mycelium or mycelial extract from *A. camphorata* has been found to inhibit HSCs activation *in vitro* and CCl_4_ or thioacetamide induced liver fibrosis *in vivo* (Schyman et al., 2019). Two maleimide derivatives Antrodin B and Antrodin C isolated from the mycelia of *A. camphorata* inhibited the growth of Lewis lung carcinoma cells *in vitro* (Huang et al., 2019). Antrodin C inhibited breast cancer cell migration and invasion by suppressing Smad2/3 and β-Catenin signaling pathways (Kumar et al., 2015). We previously identified Antrodin B from *A. camphorata* as a novel anti-fibrotic compounds through a bioassay-guided fractionation approach (Geng et al., 2016). Antrodin C also suppressed lipopolysaccharide-induced inflammation (Lee et al., 2014). However, whether Antrodin C could inhibit HSCs activation and liver fibrosis, and the underlying mechanism remains unclear.

In this study, we isolated Antrodin C from mycelial extract of *A. camphorata* and investigated the effect and potential mechanism of Antrodin C on the aHSCs *in vitro* and CCl_4_ induced liver fibrosis in mice. Together, we unraveled Antrodin C as an active compound in *A. camphorata* against liver fibrosis.

## Materials and Methods

### Chemicals

3- (4, 5-dimethyl-2-thiazolyl)-2, 5-diphenyl-2H-tetrazolium bromide (MTT) (88417), silybin (S0292), SB431542 (616464), and other chemicals were purchased from Sigma-Aldrich (St. Louis, MO, United States). Recombinant Human TGF-β1 (AF-100-21C), and PDGF-BB (AF-100-14B) were purchased from PeproTech (Rocky Hill, NJ, United States). RPMI 1640 medium, fetal bovine serum (FBS), trypsin and antibiotics (penicillin and streptomycin) were purchased from Gibco (Thermofisher, USA).

### Isolation and Identification of Antrodin C

The extraction and fractionation procedures were described previously (Geng et al., 2016). Briefly, the dried mycelium of *A. camphorata* was extracted in methanol and then partitioned with n-hexane (319902, Sigma-Aldrich), chloroform (288306, Sigma-Aldrich) and ethyl acetate (319902, Sigma-Aldrich). The n-hexane-soluble fraction was chromatographed on a silica gel column eluted with a gradient of n-hexane and ethyl acetate. The fraction eluted by 16% of EtOAc was further separated by a semipreparative HPLC column (Waters XBridge C18 column, Ф19 × 250 mm, 5 μm) (Supplementary Figure S1). The mobile phase consisted of distilled water H_2_O (0.5% acetic acid) and acetonitrile (34851, Sigma-Aldrich) at 10 ml/min to obtain Antrodin C (purity > 95%). The structures of Antrodin C was analyzed by comparing their LC-MS, ^1^H, ^13^C NMR spectroscopic data (Supplementary Figures S2–S4) and compared with published data (Nakamura et al., 2004).

### Cell Culture

Rat hepatic stellate cell line (CFSC-8B cells) were obtained from the cell bank of Xiangya Central Experiment Laboratory of Central South University (Changsha, China). CFSC-8B cells were cultured at 37°C in a humidified 5% CO_2_ incubator and RPMI 1640 medium, supplemented with 10% FBS, 100 U/mL penicillin and 100 mg/ml streptomycin.

### Cell Viability

The viability of CFSC-8B cells was quantified by the ability of living cells to reduce the yellow dye MTT to a blue formazan product. CFSC-8B cells (8 × 10^4^ cells/mL) were seeded in a 96-well cell culture plate (Corning Incorporated, USA) and grew to 80%–90% confluence. Then the cells were incubated with Antrodin C (10–200 μM) for 24 h. The viability (% of the control) of cells treated with Antrodin C was calculated as 100% × (absorbance of treated cells/absorbance of control cells).

### Cell Migration Assay

CFSC-8B cells (3 × 10^6^ cells/mL) were seeded onto the upper chambers (8 μm pore size, Milipore, Billerica, Massachusetts, United States) with 100 μL of 0.5% FBS medium and 0.5 ml normal growth medium was added to the lower chambers as a chemoattractant in 24 well plate for 24 h. The cells left on the upper chambers were removed using a cotton swab. Then the chambers were fixed using 4% paraformaldehyde for 30 min, washed and stained 0.5% crystal violet for 30 min at 37°C. Five random views were photographed under a microscope (Nikon, Tokyo, Japan). The positive cells were counted and quantified using ImageJ. Silybin (25 μM) and SB431542 (2 μM) were used as positive controls in the assay.

### Animals and CCl_4_ Induced Liver Fibrosis

All animal experiments were approved by the Animal Research Committee of Jiangnan University. Male 6–8 weeks old BALB/c mice were obtained from Shanghai SLAC Laboratory Animal Co., Ltd. The animals were housed under standard conditions and fed with a normal chow diet (M01-F25-20150922034, Shanghai SLAC Laboratory Animal Co., Shanghai, China). The mice were randomly and equally divided into the CTL group, Silymarin group, CCl_4_ group and two Antrodin C treatment groups (*n* = 6 per group). In the Antrodin C treatment groups, mice were orally administered with Antrodin C (3 or 6 mg/kg/d, formulated in 0.5% Carboxymethylcellulose sodium) daily for 2 weeks before CCl_4_ injection. Silymarin was used as a positive drug control at the dose of 100 mg/kg/d daily for 2 weeks before CCl_4_ injection. Then the CCl_4_, Silymarin and Antrodin C groups were intraperitoneal injection CCl_4_ (0.5 ml/kg, 25% solution in olive oil) twice per week, and the control group was given the same dose of olive oil. The Silymarin or Antrodin C groups were orally administered with Antrodin C or Silymarin for 4 weeks together with CCl_4_ injection.

### Histology Analysis

Liver tissues were embedded in paraffin and 4-μm-thick slices were cut, and placed on glass slides. Slides were stained with hematoxylin-eosin (H&E) or Sirius-red, and examined under a light microscope (Nikon, Tokyo, Japan). HE staining was performed to assess pathological changes in the liver. Sirius-red staining was performed to detect collagen deposition and was analyzed by ImageJ software (NIH, Bethsda, MD).

### Measurement of Serum Aminotransferase Activities and Hydroxyproline Contents

The activities of alanine aminotransferase (ALT), aspartate aminotransferase (AST) in serum, and hydroxyproline contents in liver tissues were estimated spectrophotometrically using commercial diagnostic kits (Jiancheng Institute of Biotechnology, Nanjing, China).

### RNA Isolation and qRT-PCR Analysis

Total RNA was extracted from mouse liver tissue or cells with Trizol reagent (Thermofisher, CA, United States). Gene expressions were measured relative to the endogenous control gene Gapdh using the comparative CT method and the sequences of specific primer pairs for α-SMA, Col1, and Col3 were described previously (Geng et al., 2016).

### Western Blotting Analysis

Protein extracted from cells was resolved by SDS-polyacrylamide gel electrophoresis and transferred to PVDF membranes. Antibodies against α-SMA, GAPDH were from Sigma-Aldrich (St. Louis, MO, United States). Antibodies against Smad2, phospho-Smad2, P38, phospho-P38, ERK, phospho-ERK, AKT, phospho-AKT were purchased from Cell Signaling Technology (Danvers, MA, United States). The bands were visualized using ECL reagents (Pierce, Thermofisher Scientific, USA). Band intensity was quantified using Image lab software (Bio-Rad Laboratories, Inc. USA) and expressed as relative intensity compared with control. GAPDH level served as an internal control.

### Statistical Analysis

Data are expressed as means ± SD. Differences in measured variables among groups were assessed by using One-way analysis of variance (ANOVA), and the Tukey test was used for determining the significance (Graphpad, San Diego, CA, United States). Results were considered statistically significant at *p* < 0.05.

## Results

### Effect of Antrodin C on the Survival of CFSC-8B Cells

**FIGURE 1:**
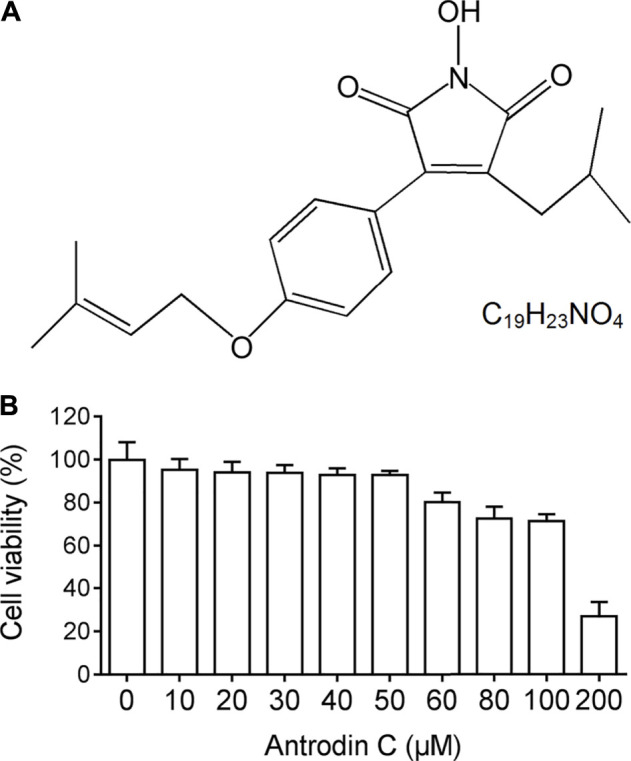
The chemical structure and cell viability of CSFC-8B cells treated with Antrodin C **(A)** The chemical structure of Antrodin C. **(B)** Effect of Antrodin C on cell viability of CFSC-8B cells by MTT assay.

We examined the cytotoxic effects of Antrodin C (Figure 1A) on hepatic stellate CSFC-8B cells using an MTT assay. CFSC-8B cells were treated with 10–200 μM Antrodin C for 24 h (Figure 1B). When the concentration of Antrodin C was above 60 μM, the cell survival rate was less than 80%. Antrodin C at the concentration of 200 μM significantly inhibited CFSC-8B cell proliferation with 82% inhibition. The median inhibitory concentration (IC50) of Antrodin C for CFSC-8B cells was 147.91 μM. The data showed that Antrodin C is able to reduce the proliferation of HSCs, and because we found that the concentrations below 50 μM of Antrodin C do not have significant cytotoxic effects on CFSC-8B cells, we used these concentrations (12, 25, 50 μM) in the following experiments.

### Antrodin C Inhibits TGF-β1 Induced Cell Migration in CFSC-8B Cells

**FIGURE 2:**
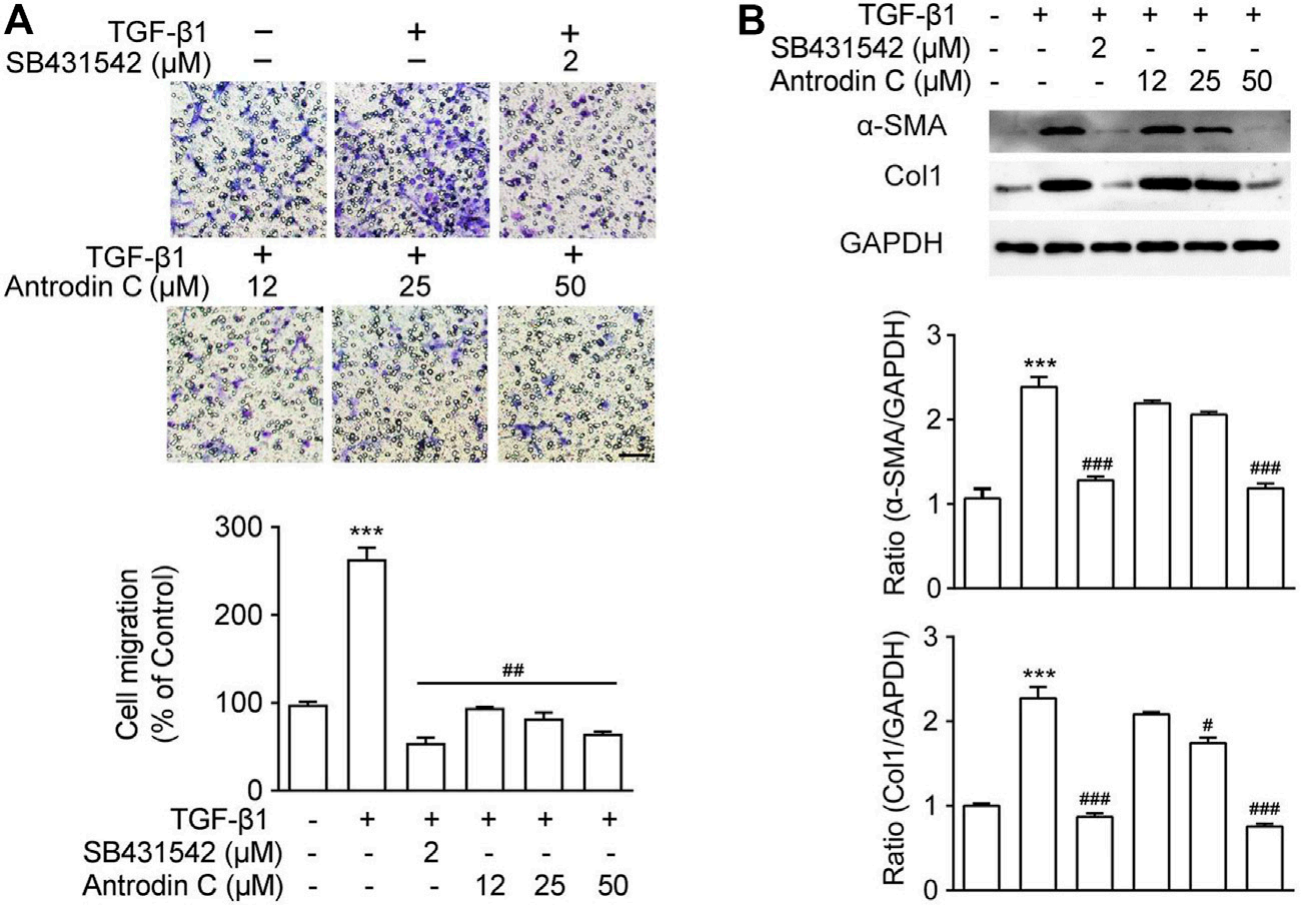
Effects of Antrodin C on TGF-β1 induced cell migration as well as α-SMA and Col1 production in CFSC-8B cells. **(A)** Representative images of migrated CSFC-8B cells treated with or without Antrodin C (12–50 μM) and SB431542 in the presence or absence of TGF-β1. The data were normalized to % of migrated control cells. Scale bar, 100 μm. **(B)** Western blot analysis of α-SMA and Col1 expression. GAPDH was used as an internal control. Data represent mean ± SD (*n* = 3), ***p < 0.001 compared with control; #*p* < 0.05, ##*p* < 0.01, ###*p* < 0.001 compared with cells treated with TGF-β1 only.

A previous study suggested that the cell migration of aHSCs is involved in the initial pathological development of liver fibrosis (Selenina et al., 2019). As anticipated, TGF-β1 (10 ng/ml) up-regulated CFSC-8B cell migration, while TGF-β receptor inhibitor SB431542 (Geng et al., 2016) dramatically decreased TGF-β1 induced cell migration (Figure 2A). Antrodin C (12–50 μM) inhibited cell migration stimulated by TGF-β1 in a dose-dependent manner in CFSC-8B cells (Figure 2A). These results suggest that Antrodin C might play an inhibitory role in TGF-β1-mediated HSCs activation.

### Antrodin C Reduces Activation and ECM Accumulation Induced by TGF-β1 in CFSC-8B Cells

To reveal the role of Antrodin C in HSCs activation, we assessed the effect of Antrodin C intervention on TGF-β1 induced expression of α-SMA and collagen I, that were the markers of HSCs activation (Tsuchida and Friedman, 2017). As shown in Figure 2B, TGF-β1 significantly increased a-SMA and Col1 expression in CFSC-8B cells. Antrodin C at a concentration of 50 μM suppressed a-SMA protein expression. The production of Col1 was also suppressed by the addition of Antrodin C (12–50 μM) in a dose-dependent manner (Figure 2B). Similar results were observed at the gene expression of a-SMA and Col1 by qRT-PCR analysis (Supplementary Figure S5A). Furthermore, Antrodin C (25–50 μM) inhibited TGF-β1 induced Col3 and Fibronectin (Fn) transcription in CFSC-8B cells (Supplementary Figure S5A). These data indicate that Antrodin C can inhibit the TGF-β1 induced HSCs activation and ECM production.

### Antrodin C Suppresses TGF-β1-Stimulated Phosphorylation of Smad2, AKT, ERK, and P38 in CFSC-8B Cells

**FIGURE 3:**
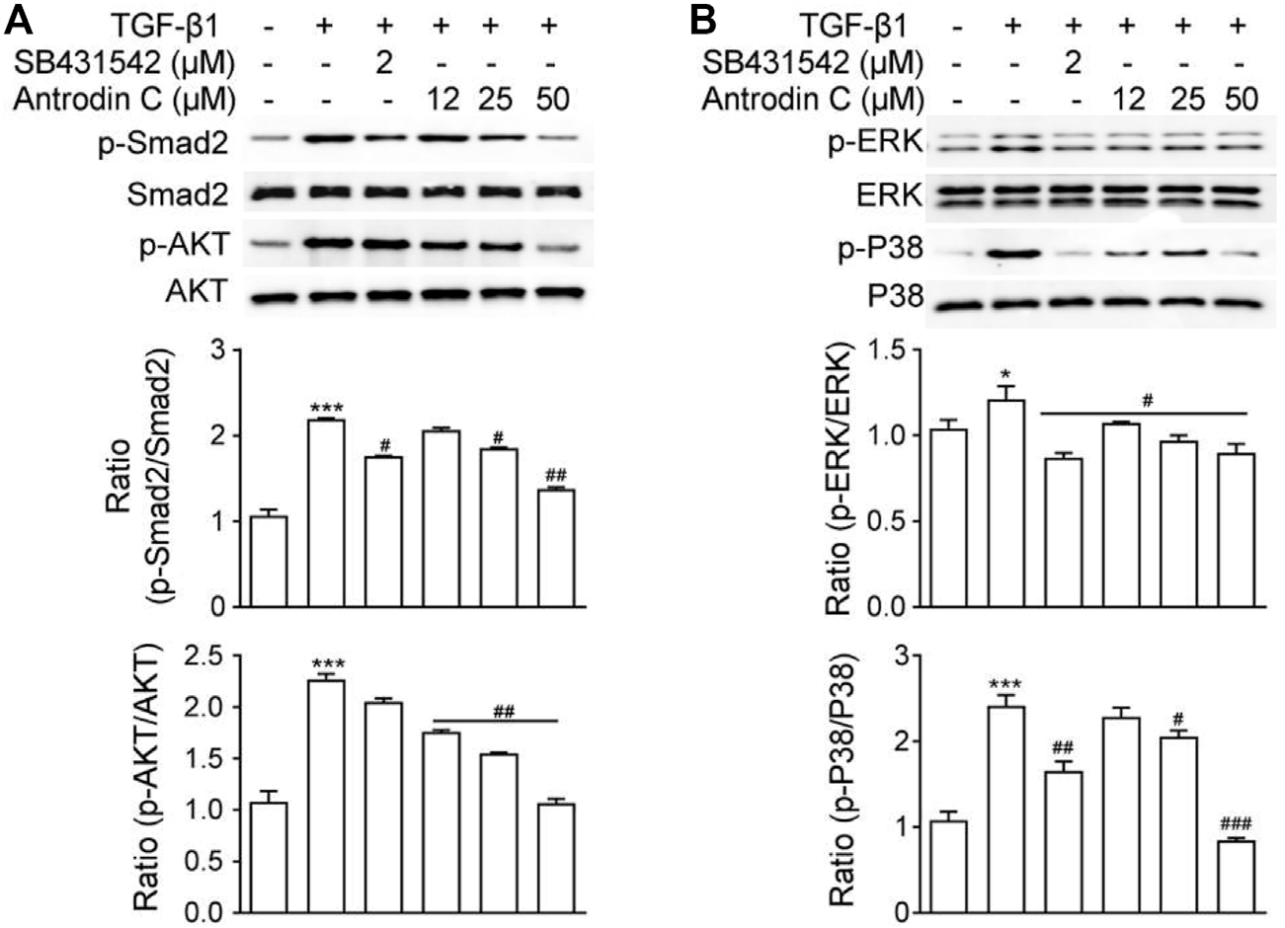
Effects of Antrodin C on the TGF-β1-induced signaling pathway. **(A,B)** CFSC-8B cells were treated with Antrodin C (12–50 μM) for 2 h and then induced by TGF-β1 for 1 h, and total cellular extracts were prepared and subjected to Western blot analysis to measure the levels of phosphorylated Smad2, AKT, ERK, and P38. Total Smad, AKT, ERK, and P38 were used for normalization. Data represent mean ± SD (*n* = 3), *p < 0.05, ***p < 0.001 compared with control; #*p* < 0.05, ##*p* < 0.01, ###*p* < 0.001 compared with TGF-β1 treated only.

TGF-β1 is a well-known fibrogenic cytokine that activates HSCs and induces ECM production. Classical TGF-β1 signaling is initiated with ligand-induced oligomerization of serine/threonine receptor kinases, then through phosphorylation of the cytoplasmic signaling molecules Smad2 and Smad3 (Zhao et al., 2020). As expected, TGF-β1 stimulated p-Smad2 expression in CFSC-8B cells, which was blocked by TGF-β1 receptor inhibitor SB431542 (Figure 3A). Within the safe doses (25–50 μM), treatment with Antrodin C reversed TGF-β1 induced p-Smad2 (Figure 3). TGF-β signaling can also modulate Smad-independent pathways, including PI3K/AKT, ERK, JNK, and p38 MAPK pathways (Suwanabol et al., 2012). We found that TGF-β1 significantly up regulated the phosphorylation of AKT, ERK and P38 in CFSC-8B cells (Figure 3). Antrodin C treatment dose dependently inhibit TGF-β1 induced phosphorylation of AKT, ERK, and P38 (Figure 3). These results suggest that Antrodin C not only inhibits the classical TGF-β1 pathway, but also suppresses non-classical TGF-β1 signaling in HSCs activation, which is characterized by decreased TGF-β1 induced phosphorylation level of Smad2, AKT, ERK, and P38.

### Antrodin C Inhibits PDGF-BB-Induced Cell Migration in CFSC-8B Cells

**FIGURE 4:**
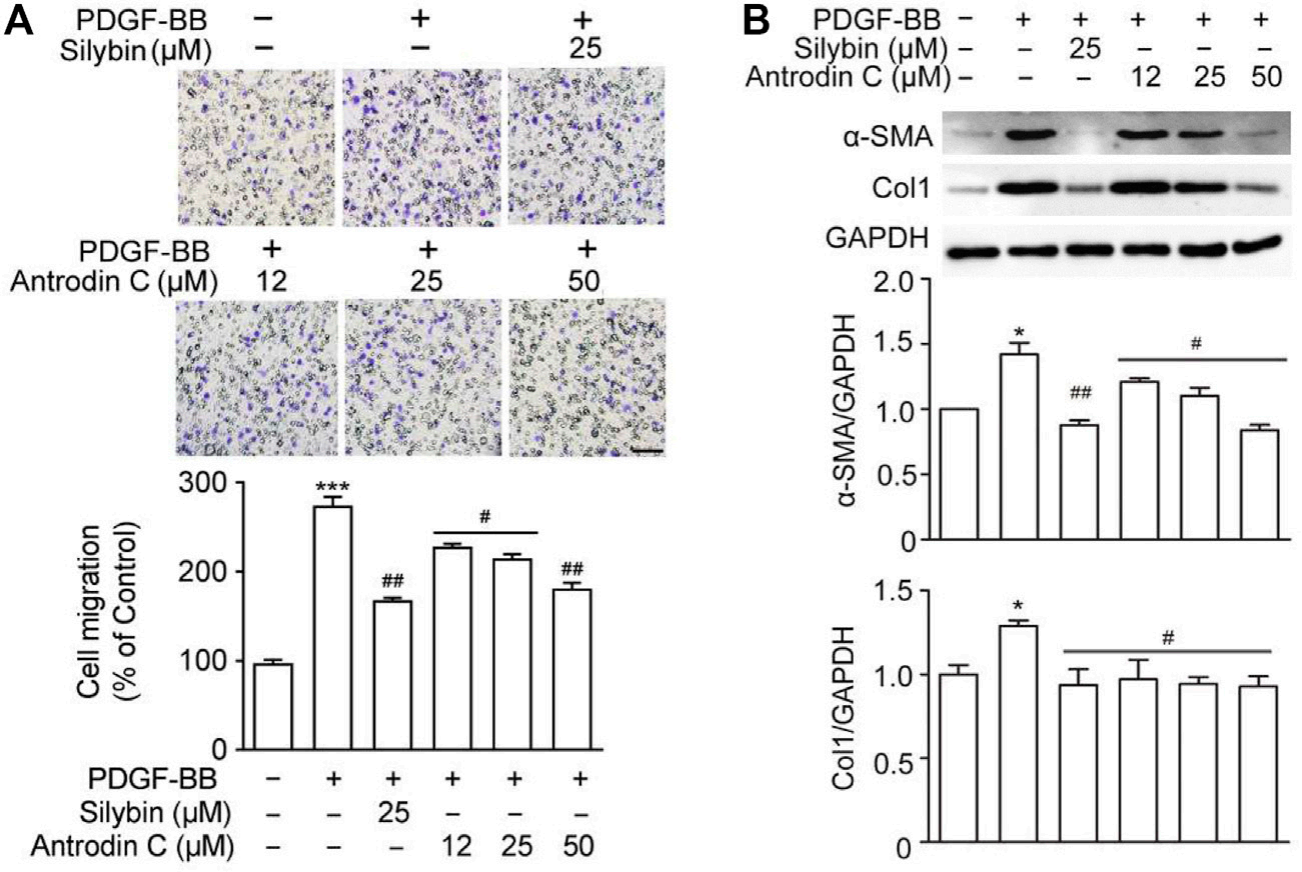
Effects of Antrodin C on PDGF-BB induced cell migration, α-SMA and Col1 production in CFSC-8B cells. **(A)** Representative images of migrated CSFC-8B cells treated with or without Antrodin C (12–50 μM) and Silybin in the presence or absence of PDGF-BB. The data were normalized to % of migrated control cells. Scale bar, 100 μm. **(B)** Western blot analysis of α-SMA and Col1 expression. GAPDH was used as an internal control. Data represent mean ± SD (*n* = 3), ***p < 0.001 compared with control; #*p* < 0.05, ##*p* < 0.01, ###*p* < 0.001 compared with PDGF-BB treated only.

The presence of PDGF-BB accelerated HSCs migration by chemoattractant mechanism (Ikeda et al., 2010). We found that PDGF-BB (10 ng/ml) induced cell migration (Figure 4A), while the positive control Silybin (25 μM) which is the major active constituent of hepatoprotective and anti-fibrotic drug, inhibited this effect (Brinda et al., 2012). Antrodin C (12–50 μM) suppressed the migration of CFSC-8B cells stimulated with PDGF-BB (10 ng/ml) in a dose-dependent manner (Figure 4A).

### Antrodin C Reduces HSC Activation and ECM Accumulation Induced by PDGF-BB in CFSC-8B Cells

We then examined whether Antrodin C was able to inhibit the HSCs activation and ECM production induced by PDGF-BB at the molecular level. The remarkable makers of HSCs activation α-SMA and Col1 were both significantly induced by PDGF-BB treatment (Figure 4B). These inductions were suppressed by the addition of Silybin (25 μM) as well as Antrodin C (12–50 μM) (Figure 4B). Furthermore, α-SMA, Col1, Col3, and Fn transcription levels were significantly decreased in PDGF-BB stimulated CFSC-8B cells after 50 μM Antrodin C treatment (Supplementary Figure S5B). Thus, Antrodin C inhibits PDGF-BB induced HSCs activation and ECM production.

### Antrodin C Treatment Downregulates PDGF-BB-Induced ERK and Akt Phosphorylation in CFSC-8B Cells

**FIGURE 5:**
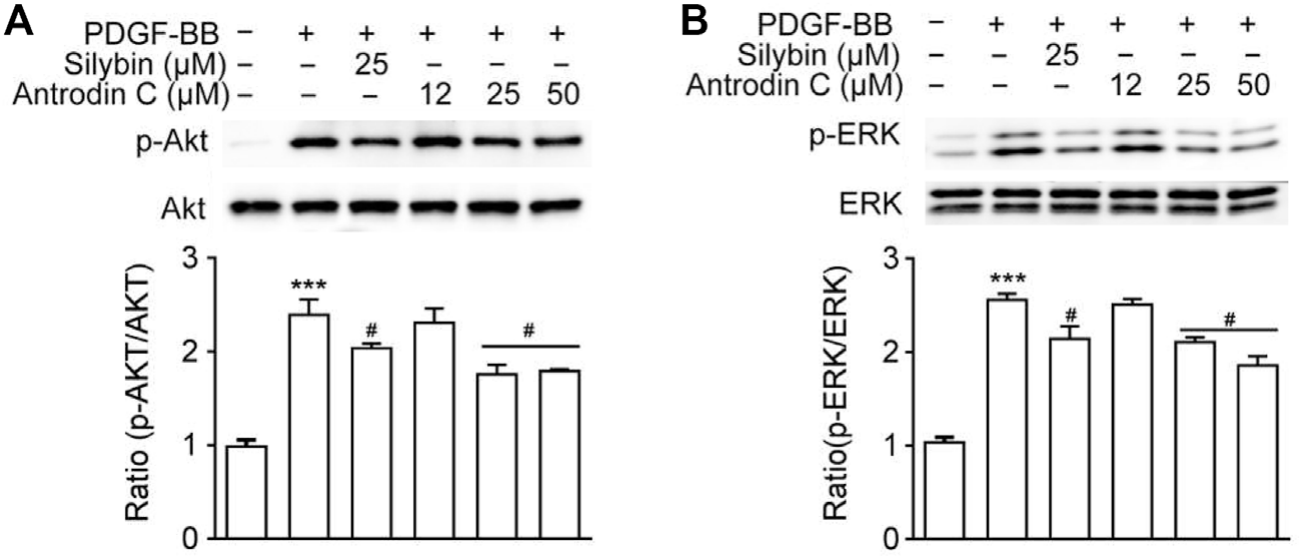
Effects of Antrodin C on the PDGF-BB-induced signaling pathway. **(A,B)** CFSC-8B cells were treated with Antrodin C (12–50 μM) for 2 h and then induced by PDGF-BB for 1 h, and total cellular extracts were prepared and subjected to Western blot analysis to measure the levels of phosphorylated AKT and ERK. Total AKT and ERK were used for normalization. Data represent means ± SD (*n* = 3), ***p < 0.001 compared with control; #*p* < 0.05 compared with PDGF-BB treated only.

PDGF-BB is one of the most potent HSC mitogen, which binds to PDGFR-β, then sequentially activates Raf-1, MEK and extracellular-signal regulated kinase (ERK). PDGF also induces PI3K/Akt signaling pathway, which is necessary for both mitogenesis and chemotaxis and involved in activating the Ras-ERK pathway (Tricarico et al., 2002). As shown in Figure 5A, Antrodin C (25, 50 μM) decreased the phosphorylation of ERK compared to that of PDGF-BB-induced cells. Antrodin C (25, 50 μM) also significantly blocked PDGF-BB induced Akt phosphorylation (Figure 5B). These results demonstrate that the inhibitory effect of Antrodin C on liver fibrosis might be through inactivation of PDGF-BB induced p-ERK and p-Akt.

### Antrodin C Protects CCl_4_ Induced Liver Fibrosis in Mice

**FIGURE 6:**
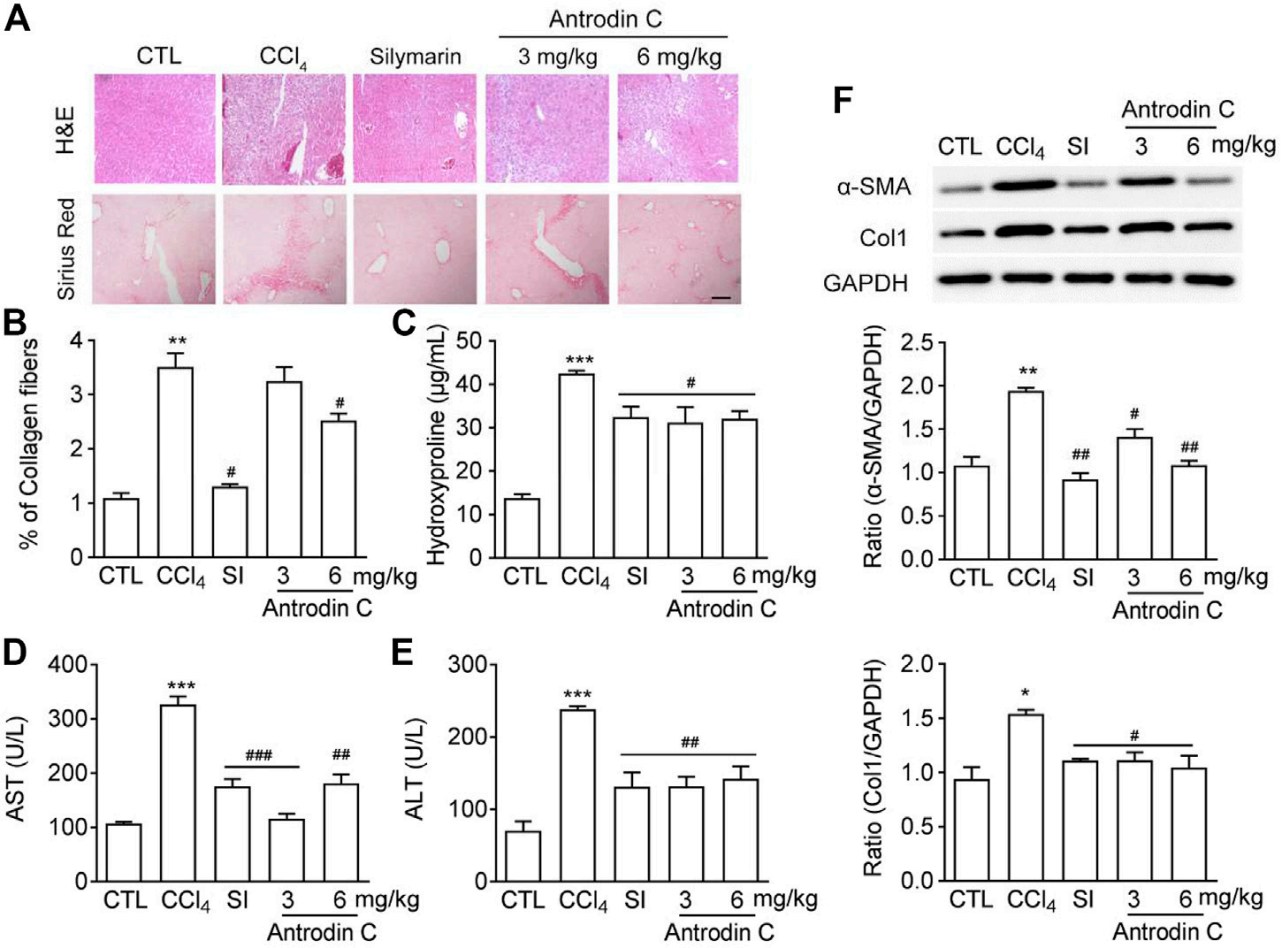
Effects of Antrodin C on liver fibrosis induced by CCl_4_ *in vivo*. **(A)** Representative images of H&E and Sirius Red staining of liver tissue sections from the different treatment groups. Scale bar, 100 μm. **(B)** The Sirius red staining was normalized to % of collagen fibers of the control group. **(C)** Hydroxyproline contents, **(D)** ALT and **(E)** AST activity in serum of CCl_4_-induced mice or control mice. **(F)** Western blot analysis of α-SMA and Col1 expression in livers tissues. GAPDH was used as an internal control. Data represent means ± SD (*n* = 6), *p < 0.05, **p < 0.01, ***p < 0.001 compared with control group; #*p* < 0.05, ##*p* < 0.01, ###*p* < 0.001 compared with CCl_4_-treated group. CTL: the control group; CCl_4_: the CCl_4_ treated group; SI: the Silymarin treated group.

To determine the role of Antrodin C *in vivo*, we used the well-established model of CCl_4_ induced liver fibrosis in mice. Histopathological analysis by H&E and Sirius-red staining showed a considerable increase in the extent of liver fibrosis after repeated injection of CCl_4_ (Figure 6A). The oral administration of Antrodin C or positive control Silymarin reduced the degree of liver fibrosis as determined by histopathological analysis as well as the quantification of Sirus-red staining (Figures 6A,B). Hydroxyproline content further confirmed that Antrodin C inhibited CCl_4_ induced collagen production in mice (Figure 6C). ALT (alanine transaminase) and AST (aspartate aminotransferase) have been recognized as indicators of liver function (Odiba et al., 2014). The elevated serum ALT and AST revealed that CCl_4_ treatment cause hepatotoxicity, while Antrodin C treatment dramatically inhibited this effect (Figures 6D,E). HSCs are mainly responsible in the progression of liver fibrosis which produces massive ECM including collagens, and α-SMA is a marker of the activated HSCs (Tsuchida and Friedman, 2017). Repeated CCl_4_ injection resulted in the liver fibrosis with increased α-SMA and Col1 protein expression (Figure 6F). Immunoblotting analysis revealed that α-SMA and Col1 were less expressed in the liver tissues from the Antrodin C treated group than the CCl_4_ group (Figure 6F). Therefore, Antrodin C showed a protective effect on liver injury and fibrosis.

### Antrodin C Inhibits CCl_4_ Induced Phosphorylation of Smad2, -Akt, -ERK and -P38 MAPK in Mice

**FIGURE 7:**
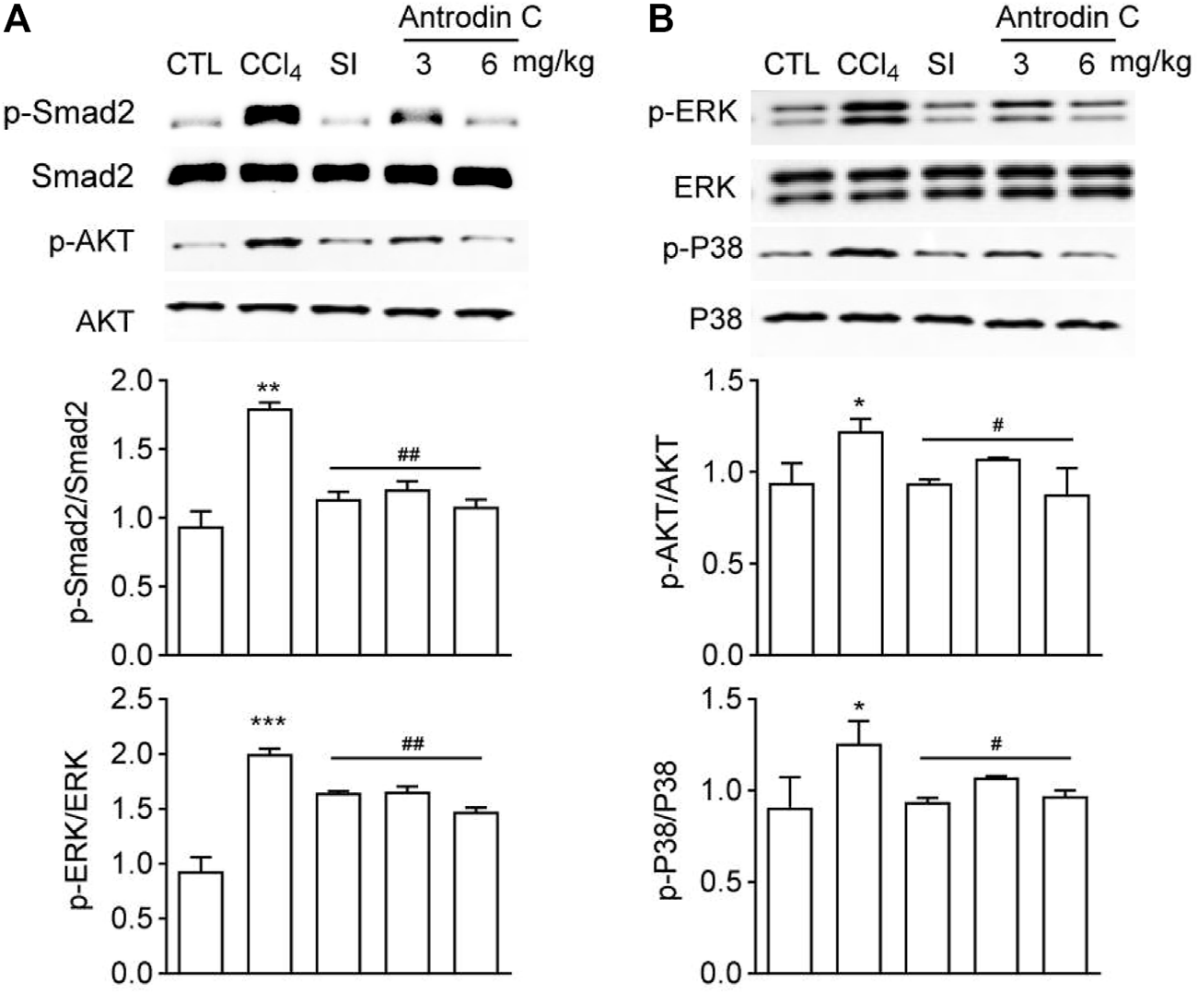
Effects of Antrodin C on phosphorylation of Smad2, AKT, ERK, and P38 in the livers of CCl_4_-treated mice. **(A,B)** Western blot analysis of phosphorylated and total Smad2, AKT, ERK, and P38 in livers tissues. Total Smad2, AKT, ERK, and P38 were used for normalization, respectively. Data represent mean ± SD (*n* = 6), *p < 0.05, **p < 0.01, ***p < 0.001 compared with control group; #*p* < 0.05, ##*p* < 0.01 compared with CCl_4_-treated group. CTL: the control group; CCl_4_: the CCl_4_-treated group; SI: the Silymarin treated group.

To explore a potential signaling pathways affected by Antrodin C treatments, we determined the levels of p-Smad2, p-Akt, p-ERK, and p-P38 MAPK, which belong to the TGF-β1 or PDGF-BB mediated signaling pathways. The results showed that the p-Smad2, p-Akt, p-ERK, and p-P38 were increased in mice treated with CCl_4_ compared with the control group (Figures 7A,B). Antrodin C treatment significantly inhibited the phosphorylation of Smad2, Akt, ERK, JNK and P38 (Figures 7A,B). These results demonstrate that Antrodin C offers a promising therapeutic strategy for patients with liver fibrosis through inhibiting p-Smad2, p-Akt, p-ERK, and p-P38.

## Discussion

Many bioactive components/compounds were identified in *A. camphorata*, including triterpenoids, polysaccharides, benzenoids, lignans, steroids, succinic, and maleic derivatives (Geethangili and Tzeng, 2010). However, little attention has been focused on why *A. camphorate* can inhibit liver fibrosis and what ingredients play a key role. In our study, we found that Antrodin C isolated from *A. camphorata* demonstrate significant anti-liver fibrosis properties. Antrodin C significantly inhibited cell migration, ECM production and HSCs activation induced by TGF-β1 or PDGF-BB in a dosage-dependent manner. In terms of mechanism, Antrodin C suppressed TGF-β1 induced p-Smad2, p-AKT, p-ERK, p-P38, as well as PDGF-BB induced p-AKT and p-ERK. We also showed that Antrodin C could attenuate CCl_4_-induced liver fibrosis in mice through blocking phosphorylation of Smad2/Akt/ERK/P38.

HSCs are believed to be a key player of liver fibrosis. Growth factors (TGF-β and PDGF) can provoke the activation of the HSCs, and activated HSCs migrate to the sites of damaged tissue and contribute to the expansion of fibrotic lesions in liver. We used CFSC-8B cells which are an immortalized rat liver stellate cell line as an *in vitro* cell model of hepatic fibrosis (Greenwel et al., 1995). We observed that Antrodin C inhibited TGF-β1 or PDGF-BB stimulated CFSC-8B cell activation, migration and ECM production.

Current anti-fibrotic therapeutic strategies include inhibition of HSCs migration, TGF-β expression and activation, blocking TGF-β canonical or non-canonical signaling pathways (Rosenbloom et al., 2013). TGF-β signaling pathways are critical for the fibrotic response. The canonical TGF-β1 signaling is mediated through receptor binding, then through receptor activating Smads, Smad2, or Smad3 (Kastanis et al., 2011). In addition to the Smad dependent pathway, TGF-β1 also activate PI3K/AKT, ERK, JNK, and P38 MAPK signaling pathways, which play an important role in regulating HSC activation and ECM synthesis (Voloshenyuk et al., 2011). Our data showed that TGF-β1 stimulated the canonical or non-canonical signaling indicated by the up regulation of phosphorylation of Smad2, Akt, ERK, and p38 MAPK, which could be inhibited by Antrodin C.

PDGF signaling plays a critical role in various cellular responses including proliferation, chemotaxis and actin reorganization. PDGFs bind and activate tyrosine kinase receptors, PDGFRα and PDGFRβ, which leads to overlapping signaling, including Src family kinases, PI3K/AKT and Ras-MAPK. PDGF can affect the fibrotic progression of several organs, including liver, lung and kidney (Steiner and Higgins, 2018). Several reports have implicated that there was a crosstalk between TGF-β1 and PDGF signaling (Novosyadlyy et al., 2006). Our results demonstrated that Antrodin C might suppress the PDGF induced activation of HSCs through blocking phosphorylation of Akt and ERK.

CCl_4_-induced hepatic fibrosis in mice is a well-known *in vivo* model for screening anti-fibrotic drugs (Kuwahata et al., 2012). The metabolites of CCl_4_ cause the apoptosis of hepatocyte and liver injury. Repeated injection of CCl_4_ causes chronic injury to the liver, eventually results in liver fibrosis. Hepatocyte integrity indicators ALT and AST were both up regulated after CCl_4_ treatment. We found that the treatment with Antrodin C significantly diminished the liver fibrosis accompanied by the decreasing AST and ALT levels in blood circulation. Antrodin C also inhibited the activation of HSC *in vivo* revealed by the decreased expression level of α-SMA. In terms of mechanism, it was found that repeated CCl_4_ injections could induce the phosphorylation of Smad2, Akt and MAPK in the mouse liver tissues (Yoshida et al., 2005), whereas the oral administration with Antrodin C suppressed the phosphorylation of Smad2, Akt, ERK and P38 in the fibrotic livers. Further studies are needed to identify the underlying receptor-dependent. Moreover, Antrodin C may inhibits liver fibrosis by other Signaling pathways like TNF-α-NF-κB, AMPK, and β-Catenin Signaling pathway (Yang et al., 2019).

In conclusion, Antrodin C suppresses HSC activation, migration and ECM production, partially through inhibition of PDGF and TGF-β1 signaling pathways, that are the two most potent stimuli of liver fibrosis. Our study provides new insights for the development of therapeutic drugs against liver fibrosis. However, further studies are needed to fully understand the cellular and molecular mechanisms of Antrodin C in liver fibrosis.

## Data Availability

The original contributions presented in the study are included in the article/Supplementary Material, further inquiries can be directed to the corresponding authors.
