# Supplementary material for: Antrodia camphorata-Derived Antrodin C Inhibits Liver Fibrosis by Blocking TGF-Beta and PDGF Signaling Pathways
Source: Front Mol Biosci. 2022 Feb 15;9:835508. doi: 10.3389/fmolb.2022.835508 (PMC8886226; doi:10.3389/fmolb.2022.835508)
Supplement: Supplementary file 2 [file DataSheet1.PDF]

Method Name: wj6
Run Name: 2013-03-23_23-50-37wwj6
Run Date: 2013-03-24 00:02

Column: Reveleris® Silica 12g
Flow Rate: 15 mL/min
Equilibration: 5.0 min
Run Length: 122.0 min
Air Purge Time: 0.5 min

Slope Detection: Off
ELSD Threshold: N/A
UV Threshold: 0.02 AU
UV1 Wavelength: 254 nm
UV2 Wavelength: N/A

Collection Mode: Collect None
Per-Vial Volume: 20 mL
Non-Peaks: 20 mL
Injection Type: Manual

ELSD Carrier: N/A
Solvent A: Hexane
Solvent B: Ethyl acetate
Solvent C: <No solvent chose
Solvent D: <No solvent chose

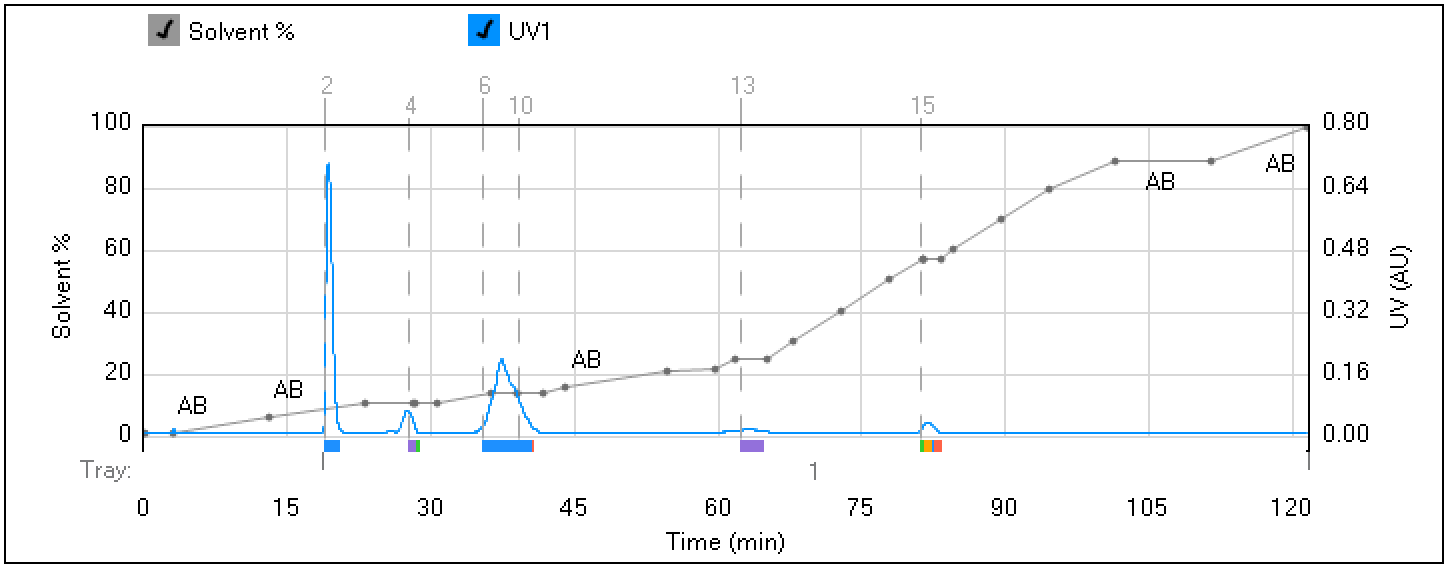

1 - EE8E

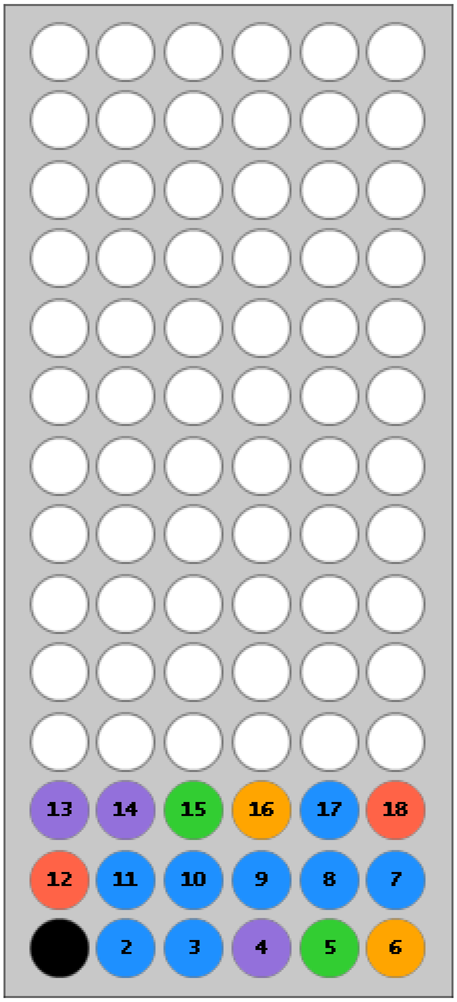

Gradient Table

	Min	Solvents	% 2nd
1	0.0	AB	0
2	3.0	AB	0
3	10.0	AB	5
4	10.0	AB	10
5	5.2	AB	10
6	0.2	AB	10
7	2.3	AB	10
8	5.7	AB	13
9	2.8	AB	13
10	0.1	AB	13
11	2.7	AB	13
12	2.2	AB	15
13	10.8	AB	20
14	5.0	AB	21
15	2.1	AB	24
16	3.3	AB	24
17	2.9	AB	30
18	5.0	AB	40
19	5.0	AB	50
20	3.5	AB	57
21	0.1	AB	57
22	0.0	AB	57
23	1.7	AB	57
24	1.5	AB	60
25	5.0	AB	70
26	5.0	AB	80
27	7.0	AB	89
28	10.0	AB	89

Gradient Table

	Min	Solvents	% 2nd
29	10.1	AB	100

Vial Mapping Table

Peak #	Start Tray:Vial	End Tray:Vial
1	1:2	1:3
2	1:4	1:4
3	1:7	1:11
4	1:16	1:16
5	1:17	1:17
