# Supplementary material for: Antrodia camphorata-Derived Antrodin C Inhibits Liver Fibrosis by Blocking TGF-Beta and PDGF Signaling Pathways
Source: Front Mol Biosci. 2022 Feb 15;9:835508. doi: 10.3389/fmolb.2022.835508 (PMC8886226; doi:10.3389/fmolb.2022.835508)
Supplement: Supplementary file 3 [file DataSheet2.docx]

Supplementary Material

**Identification of isolated compound**

The HPLC, LC-MS, ^1^HNMR and ^13^CNMR methods were used to elucidate the structures of the compound. The obtained LC-MS and NMR spectral data (Fig.S1-S4) and physicochemical properties were given below.

A yellow oily liquid, molecular formula: C_6_H_6_O_3_; EI-MS *m/z*: 330.2 [M+H]^+^; 1H-NMR(400 MHz，MeOD) δ：7.56 (d，*J*=12.0, 2H), 7.04(d，*J*=8.0，2H), 5.50 (m，1H), 4.62 (d，*J*=8.0 Hz，2H), 2.55(d，*J*=8.0 Hz,2H), 2.03 (m, 1H), 1.81(d, *J*=12.0 Hz, 6H), 0.91(d，*J*=4.0 Hz，6H); ^13^C-NMR (100 MHz, MeOD) *δ*：168.7, 167.9, 160.2, 137.7, 135.5, 130.7, 121.0, 119.4, 114.5, 64.6, 32.3, 27.9, 24.5, 21.6, 16.8; By analyzing the obtained spectral data, the compound was identified as Antrodin C.

Antrodin C

**Fig.S1** The HPLC of n-hexane-soluble fraction of *Antrodia camphorata.*


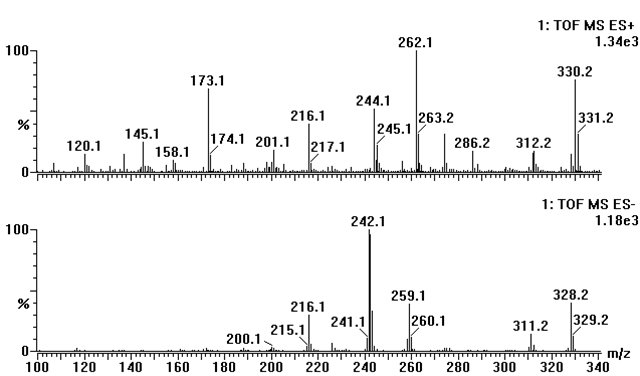


**Fig.S2** The LC-MS of the isolated compound Antrodin C

**Fig.S3** The ^1^H-NMR of the isolated compound Antrodin C

**Fig.S4** The ^13^C-NMR of the isolated compound Antrodin C

**
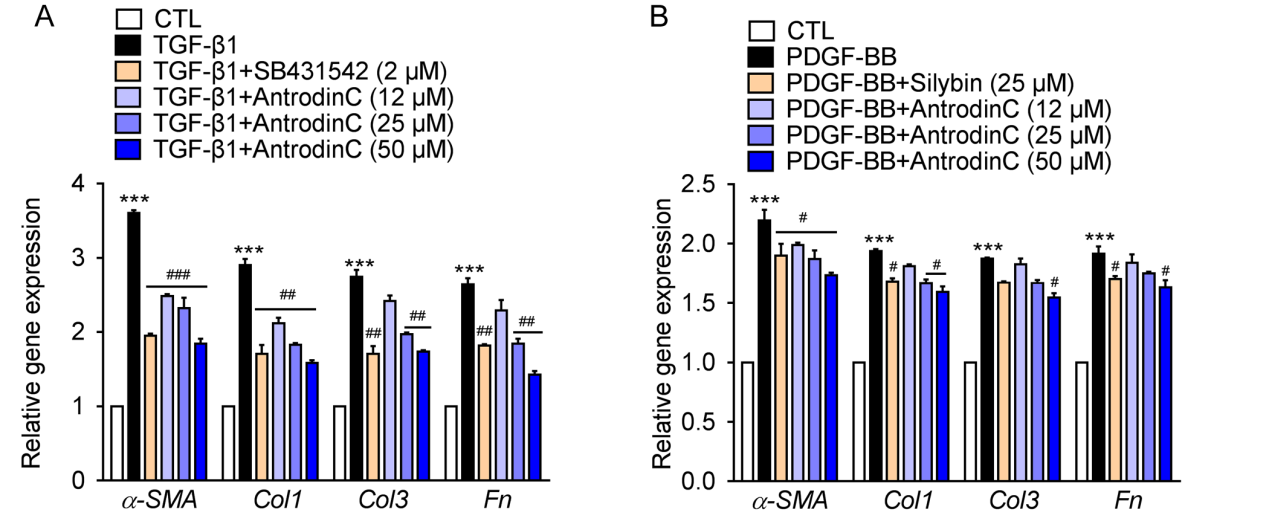
Fig. S5** Effect of Antrodin C on a-SMA, Col1, Col3 and Fn gene expression in (A) TGF-β1 or (B) PDGF-BB treated CFSC-8B cells. CFSC-8B cells were treated with Antrodin C (12-50 μM) for 2 h and then induced by TGF-β1 for 24h. SB431542 or Silybin were used as the positive control, respectively. Throughout, data represent means ± SD (n = 3), ***P < 0.001 compared with control; #P < 0.05, ##P < 0.01, ###P < 0.001 compared with TGF-β1 or PDGF-BB treated only.
